# Distribution of 5-HT_1F_ Receptors in Monkey Vestibular and Trigeminal Ganglion Cells

**DOI:** 10.3389/fneur.2016.00173

**Published:** 2016-10-10

**Authors:** Habiba O. Usman, Carey D. Balaban

**Affiliations:** ^1^Department of Otolaryngology, University of Pittsburgh, Pittsburgh, PA, USA; ^2^Department of Neurobiology, University of Pittsburgh, Pittsburgh, PA, USA; ^3^Department of Communication Sciences and Disorders, University of Pittsburgh, Pittsburgh, PA, USA; ^4^Department of Bioengineering, University of Pittsburgh, Pittsburgh, PA, USA

**Keywords:** serotonin receptors, ganglion cells, vestibular nerve, trigeminal ganglion, migraine

## Abstract

**Background:**

Evidence of serotonergic involvement in vestibular pathway contributions to migraine and balance disorders is compelling. Serotonergic 5-HT_1B_ and 5-HT_1D_ receptors are expressed extensively in inner ear ganglia of monkeys and rats. The serotonergic 5-HT_1F_ receptor is also a target of triptans. This study describes its distribution in vestibular and trigeminal ganglia of monkeys.

**Methods:**

Using primary polyclonal antibodies raised against oligopeptides specific for the human 5-HT_1F_ receptor, neuronal somatic area and intensity of immunoreactive vestibular and trigeminal ganglia were quantified.

**Results and Discussion:**

Virtually all vestibular and considerable trigeminal ganglia showed positive 5-HT_1F_ receptor immunoreactivity. Inferior and superior vestibular ganglia staining appeared confined to distinct cell regions, varying considerably among cells of different sizes: more intense in small, punctate in some medium and regionally polarized in some large cells. Analyses of average somatic vestibular neuronal immunoreactive intensity identified mainly medium sized cells with high standard deviation of intensity corresponding to punctately stained cells. Less variability occurred in somatic intensity staining and cellular distribution among 5-HT_1F_ receptor immunopositive trigeminal ganglia. Most exhibited similar punctate staining patterns, higher mean somatic immunoreactive intensity and larger neuronal somatic size proportions per size distribution subpopulation compared to vestibular ganglia size distribution populations. Centrally directed vestibular ganglion neuronal processes, cochlear inner hair cells, vestibular hair cells and blood vessels in vestibular maculae and cristae were immunoreactive. The 5-HT_1F_ receptor expression in vestibular ganglia shows complex variable staining intensity patterns associated with cell size of immunopositive neurons, not seen in immunopositive trigeminal ganglia and not previously evident with 5-HT_1B_ and 5-HT_1D_ receptor subtype immunoreactivity in vestibular ganglia. These data motivate exploration of 5-HT_1_ receptor oligomerization and ligand functional selectivity in differential serotonergic involvement in co-morbidity of migraine and balance disorders. Similar findings in cochlear inner hair cell afferents are applicable to migraine-related tinnitus or hypercusis (phonophobia).

## Introduction

The co-morbidity of migraine and balance disorders with psychiatric disorders has been well documented (1–6). One hypothesis proposed regarding these comorbidities phenomenon cites an apparent convergence of afferent channels of vestibular and trigeminal ganglia in shared central pathways for vestibular and nociceptive signal transduction (7). Numerous reports (8–12) support the concept that otologic features of vertigo associated with migraine are attributable to parallel activation of vestibular and cranial nociceptive pathways (7).

In the light of the convincing evidence of vestibular symptoms with an undeniable migrainous etiology, the International Headache Society (IHS) in conjunction with the Barany Society forwarded diagnostic criteria for the condition now known as vestibular migraine (13). Vestibular migraine is currently recognized as an episodic syndrome that may be associated with migraine by the IHS (14), a clinical condition manifesting in a considerable number of migraineurs who present with vestibular symptoms (15). Based on the considerable and rapidly expanding accumulation of reports on genetic, *in vitro* cell biological, animal model as well as human clinical studies on mechanisms triggering migraine (16–22) vestibular migraine may be viewed as a variant created by the convergence of vestibular information in migraine circuits (15).

A number of more recent reports also support the proposition that serotonergic mechanisms play a pivotal role in both peripheral and central vestibular pathway contributions to migraine and balance disorders (23, 24). Not surprisingly, serotonergic mechanisms are also suggested to be involved in mechanisms of vestibular migraine (15). Vestibular and cranial nociceptive pathways do possess striking similarities in neurochemical milieu and express serotonin receptor subtypes that are known to be targets for anti-migraine drugs, such as the triptans. Furthermore, triptans feature prominently as one of the current treatments options for migraine-associated balance disorders as well as for overt vestibular migraine (23, 25–27). Although triptans, such as rizatriptan, are particularly strong agonists at serotonergic 5-HT_1B_ and 5-HT_1D_ receptors, they also have affinity for 5-HT_1A_ (28, 29) and 5-HT_1F_ (29, 30) receptors.

The serotonergic 5-HT_1B_ and 5-HT_1D_ receptors are expressed prominently in the inner ear of rats and monkeys (31). Immunoreactivity for these receptors was mainly associated with vestibular ganglion cells, spiral ganglion cells, the vestibulocochlear nerve fibers, the spiral ligament, and stria vascularis (31). Another serotonin receptor subtype that has recently generated interest in its possible application as a promising pharmacological target option in the treatment of migraine is the 5-HT_1F_ receptor. Selective 5-HT_1F_ receptor agonists would produce fewer vascular side effects and potentially provide an alternative for patients with a history of increased risk of coronary artery disease in whom the use of the less selective vasoactive triptans would be contra-indicated (27, 32). In this present study, we examine immunoreactivity for the 5-HT_1F_ receptor in macaque vestibular and trigeminal ganglia. Our findings support our proposal that the 5-HT_1F_ receptor may also act in concert with the previously identified subtypes in the vestibular ganglia as mediators of serotonergic transmission on peripheral vestibular function.

## Materials and Methods

### Ethics

The study used archival tissues from primates that were euthanized at the conclusion of physiological studies. Two of the macaques (one male and one female adult) were used in neurophysiological recording and tract tracing experiments; the other three adult macaques (two females and one male) were acute control animals from studies of lymphoid chemokine responses to inoculation with simian immunodeficiency virus (33). Body weights ranged from 5 to 7.2 kg. The earlier studies were completed under protocols (#0898008, 0004002, 01084828, and 0304558) approved by the University of Pittsburgh Institutional Animal Care and Use Committee (IACUC) and fully certified to comply with the National Institutes for Health (NIH) and the United States Department of Agriculture standards and regulations for humane animal utilization. The University of Pittsburgh maintains an OLAW approved PHS Animal Welfare Assurance and a USDA registration. All experiments were designed to minimize the number of animals used and their suffering. These tissues were obtained from euthanized animals at the conclusion of other studies.

The animals were housed in the AALAC-accredited animal facility at the University of Pittsburgh during the studies without food restriction. The room temperature is maintained at 70 ± 4 Fahrenheit. The facility uses standard stainless steel primate caging (29″ × 30″ × 33″ or 29″ × 30″ × 69″). Macaques are typically housed in pairs. Individualized enrichment is provided at the facility by specialized, professional staff; with components that include perches in the cage; forage mix, forage boards, puzzle feeders, fruits/vegetables, assorted treats, mirrors, and stainless steel hanging toys/devices that are provided within the cage. Husbandry: pans scraped or hosed daily and cages sanitized at least once every 2 weeks.

Non-human primates are fed a commercially available biscuit type diet twice daily. The quantity of biscuits offered may be adjusted up or down based on individual body condition scores. Dietary supplements of fruit, vegetables, forage mixes, and other feedstuffs are provided daily as part of the enrichment program.

### Detailed Methods

#### Monkey Tissue Preparation

Temporal bone sections were used from a total of five adult macaque monkeys of both sexes. Three adults were acute control rhesus macaques from simian immunodeficiency virus studies (33) and two adult macaques had been subjects in chronic neurophysiology and tract tracing experiments (34, 35)]. The monkeys were previously euthanized between 1999 and 2002 with pentobarbital sodium and perfused transcardially with phosphate buffered saline (pH 7.2–7.4), followed by either perfusion fixation with 4% paraformaldehyde in phosphate buffered saline or paraformaldehyde-lysine-sodium metaperiodate (PLP) fixative. Necropsies were performed immediately after completion of the perfusion (dates: 23 September 1999, 28 September 1999, 17 November 1999, 20 May 2002, and 26 September 2002). The trigeminal ganglia were removed from the skull and processed separately for paraffin embedding. Decalcification of the temporal bone was achieved by treatment with 10% formic acid followed by an overnight soak in 5% sodium sulfate following standard methods (36). The tissue was further processed through graded ethanol concentrations and xylene, embedded in paraffin, trimmed, sectioned at 7–8 microns in the mid-modiolar plane and archived until required. There are no reports in the literature concerning potential effects of brief pentobarbital exposure on the distribution of immunoreactive serotonin receptor protein subunits.

#### Immunohistochemical Procedures

**Figure 1:**
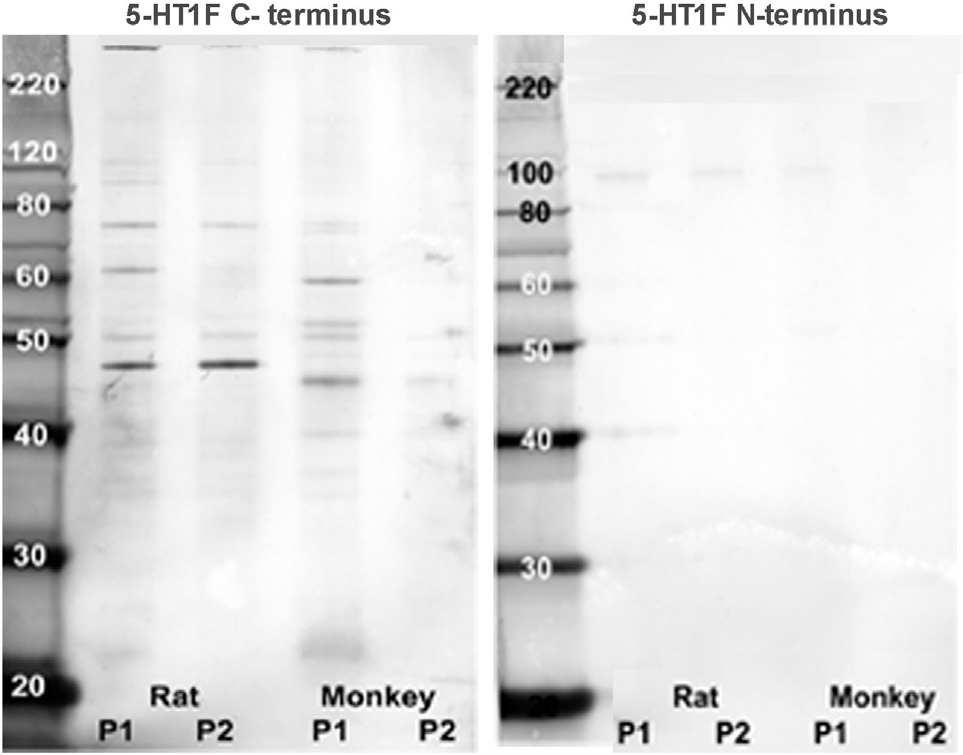
**Western blot analysis**. Rat and monkey medulla oblongata P1 and P2 fractions were prepared from homogenized fresh tissue by centrifugation. Aliquots were placed in separate lanes (labeled) for protein separation on a NuPAGE™ 10% Bis-Tris gel and were electro-blotted onto an Invitrolon™ PVDF membrane (Invitrogen). Molecular weight standards were placed in the far left lane for calibration. Binding of each primary antibody (1:1000 dilution) was detected with an alkaline phosphatase-conjugated secondary antibody and histochemical substrate (WesternBreeze™, Invitrogen). The blot was digitized with a flatbed photo scanner (Epson V500).

For the immunohistochemical procedures, the tissue sections were first de-paraffinized, rehydrated through descending ethanol concentrations to deionized water, exposed to formalin vapor, then treated for 10 min with 0.9% hydrogen peroxide in deionized water followed by thorough rinsing with deionized water. Subsequently, antigen retrieval was performed by heating the sections in a low pH (6.0) sodium citrate citric acid buffer solution for 20 min at 80–85°C followed by a thorough rinse in PBS. The sections were then treated with a blocking solution of 10% triton X-100, 2% bovine serum albumin and either normal horse or normal goat serum in PBS for 1 h at room temperature. This was followed by 24 h incubation in a polyclonal antibody raised against oligopeptides specific for the human 5-HT_1F_ N-terminus (Imgenex, 7182) or C-terminus (Imgenex, 71821). These 5-HT_1F_ antibodies had been affinity-adsorbed against their respective immunogens, synthetic oligopeptides from the N- and C-termini of the human receptor protein. Western blots of P1 and P2 fractions from rat and monkey brain tissue (Figure 1) showed a bands at between 40 and 50 kDa with the C-terminus as well as what may appear to be doublet bands at about 90 kDa particularly with the N-terminus antibody. The multiple bands with the affinity-adsorbed C-terminus antibody likely reflect the known homo- and hetero-oligimerization properties of the 5-HT1 receptor family (37–44). The primary antibodies were diluted in blocking solution (1:1000–5000). In parallel negative control experiments, the primary antibody was omitted. Following incubation in primary antibody, the sections were thoroughly rinsed with PBS and then further incubated in biotinylated secondary antibody (Jackson Laboratories, West Grove, PA, USA) diluted 1: 500 in 2% BSA in PBS for 1 h at room temperature. After three vigorous rinses to remove secondary antibody, the sections were incubated for 1 h in Vectastain ABC reagent (Vector Laboratories) and thoroughly rinsed in PBS again. Subsequently, the sections were treated with a solution of Trizma pre-set crystals (Sigma; 1.58 mg/200 ml) followed by staining with 3,3′-diaminobenzidine tetrahydrochloride (DAB stain) in sodium acetate buffer (pH 6.0). The final steps involved dehydrating the sections by successive treatment with increasing concentrations of alcohol, followed by treatment with xylene and cover-slipping with DPX.

#### Quantification Methods

Digital images of temporal bone tissue sections, stained immunohistochemically for 5-HT_1F_ receptors, were obtained with a Nikon Eclipse E600N microscope outfitted with a Spot RT Monochrome camera (Model 2.1.1, Diagnostic instruments, inc., Sterling Heights, MI, USA). The MetaMorph software (ver. 6.1r4 Universal Imaging Corporation, Downingston, PA, USA) was used to conduct the quantification of photographed images captured on a Pentium-based computer. A series of 12 bit digital images were taken with a 20× objective and the neuronal somatic area as well as intensity of 5-HT_1F_ receptor immunoreactivity of vestibular ganglia was quantified. A total of 9,991 ganglion cells were sampled from temporal bones from five monkeys. Three temporal bone sections were collected per slide and sections were sampled 350 microns apart to ensure cells were not double counted. A total of 40 slides (8 per animal) were analyzed. MetaMorph was used to manually outline and automatically log the statistics of neuronal cell bodies complete with nuclei and nucleoli. The absolute zero calibration for differences in optical transmission between the light source and CCD element were obtained by measuring areas of the images that were purely glass slide, cover slip, and mounting medium and, therefore, devoid of tissue. Readings between different slides were initially normalized by subtracting the absolute zero calibration value from recorded data. The ganglion and peripheral nerve parenchyma captured in each image had the same baseline optical density as non-immunoreactive somata and, therefore, served as control regions from which background staining intensity was obtained. Final normalization of data was achieved by subtracting the background values.

#### Statistical Analysis

The normalized data were subjected to standard statistical analyses employing SYSTAT 11 (SYSTAT Software, Inc.). The conformity of the data to assumptions of underlying normality and having identical distribution were assessed using quantile and normal probability plots. Specific hypotheses under assumptions of normality were assessed using analysis of variance (ANOVA) with least significant differences (LSD) tests. Furthermore, the distribution of any given data set as a mixture to three normal Gaussian populations was estimated by k-means cluster analysis followed by order statistics approach, achieved adhering to previously described procedure (35).

## Results

### Expression of the 5-HT_1F_ Receptor Subtype in Vestibular Periphery

**Figure 2:**
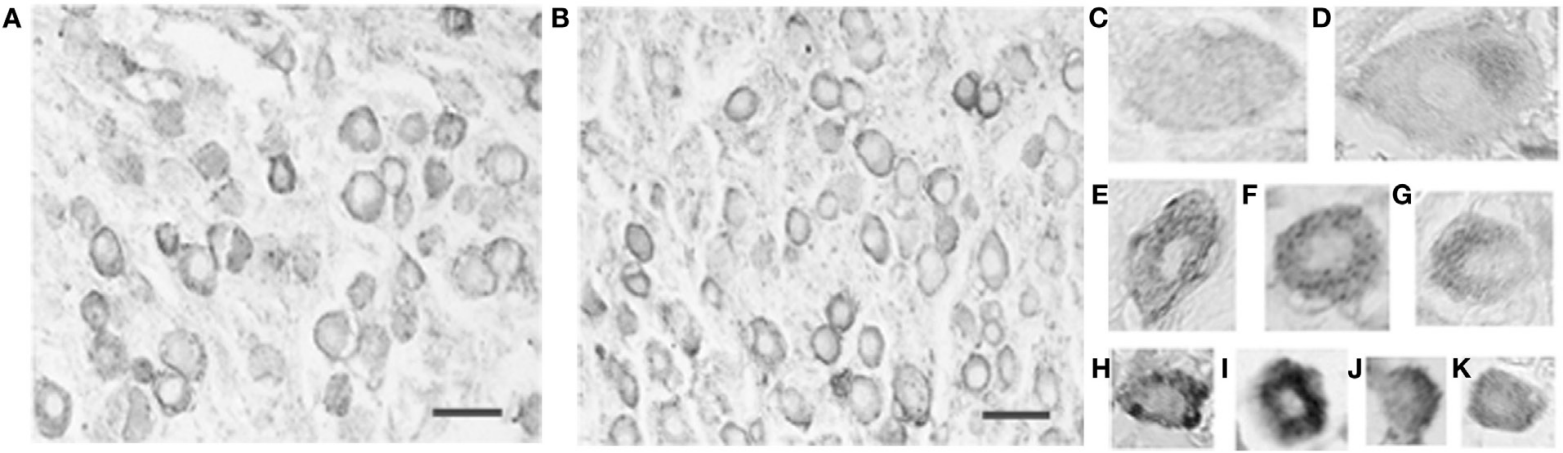
**Photomicrograph of 5-HT_1F_ receptor immunoreactive vestibular ganglion cells in the macaque**. **(A)** Inferior and **(B)** superior vestibular ganglion. Note the greater intensity of the immunoreaction in small ganglion cells and the association of immunoreactive fibers with blood vessels. The calibration bars represent 40 microns. Staining patterns and associated cell sizes in representative vestibular ganglion cells; **(C,F)** large to intermediate sized cells showing punctuate staining associated with the cell membrane. **(D,G)** Large to intermediate sized cells with demarcated immunoreactive regions at one pole of the cell. **(E,H–K)** A few intermediate and mainly small sized cells with intense, reasonably homogenous somatic immunoreactivity.

Virtually, all vestibular ganglion cells, as well as some vascular endothelial cells, were immunoreactive for the serotonergic 5-HT_1F_ receptor (Figures 2A,B) in the monkey temporal bone sections. The results were the same for the antibodies recognizing the N-terminus and C-terminus. The intensity and intracellular distribution of 5-HT_1F_ receptor immunoreactivity varied considerably among vestibular ganglion cells of different sizes. Cluster analysis indicated that the size distribution of the ganglion cells expressed as mean circular diameter (square root soma area/pi × 2) in microns is consistent with three normally distributed (Gaussian) populations consisting of 24.3% from a of 30.8 ± 6.4 (SD) micron population, while 41.2 and 26.5% belonged to populations of average circular diameters of 47.4 ± 4.6 (SD) microns and 61.2 ± 7.0 (SD) microns, respectively. Smaller sized ganglion cells tended to be stained more intensely than larger ganglion cells in both the inferior and the superior vestibular ganglia (Figures 2A,B, compare Figures 2C,D with Figures 2H,K). These included small (and a few intermediate) sized cells with intense, reasonably homogeneous somatic immunoreactivity (Figures 2H,I–K,E) as well as intermediate to large sized cells showing punctuate staining associated with the cell membrane (Figures 2C,F). In addition, some large to intermediate sized cells had strongly demarcated immunoreactive regions at one pole of the cell (Figures 2D,G).

**Figure 3:**
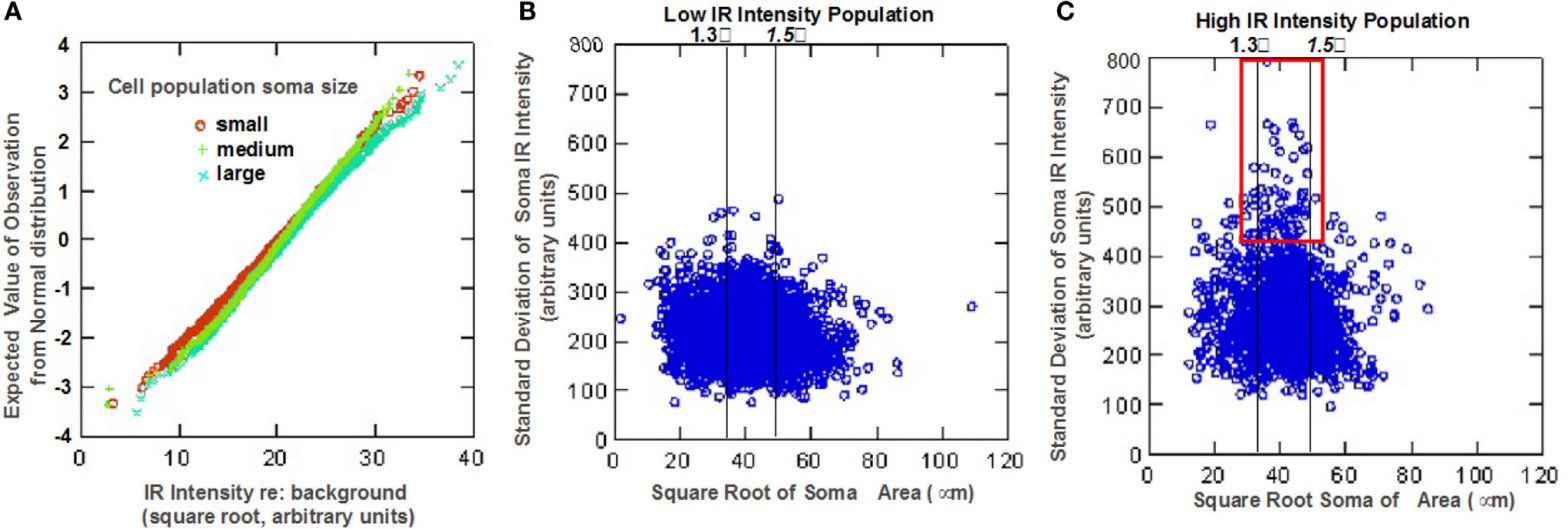
**Statistical analysis of neuronal 5-HT_1F_ receptor immunohistochemistry in vestibular ganglion cells from monkey temporal bones**. **(A)** Normal probability plots show ganglion cells with higher immunoreactive intensity tend to be larger than lower staining intensity cells. Scatter plots show **(B)** low and **(C)** high intensity staining subpopulations of the distinct neuronal staining patterns identified in Figure 2; the red square shows a subpopulation of mainly medium size cells depicted in Figure 2F.

Quantitative analysis of the somatic immunoreactivity intensity of 5-HT_1F_ positive neurons is illustrated in Figure 3. Normal probability plots of the intensity data (square root of IR intensity) for the three size intensity populations (small, medium, and large) showed that vestibular ganglion cells with higher immunoreactive intensity tended to be larger than lower staining intensity cells (Figure 3A). Cluster analysis of mean cell staining intensity (square root of mean IR intensity) identified two somatic staining intensity populations comprising 49.1% of cells with a low mean somatic intensity (17.2 ± 2.7 arbitrary units) and 50.9% of cells with a higher mean somatic intensity (23.9 ± 2.9 arbitrary units). Several interesting characteristics relating to somatic immunoreactive intensity and cell size distribution were further revealed. For example, within the subpopulation of ganglion cells with low mean somatic staining intensity, small size cells exhibited significantly less intense staining (LSD tests, p < 0.01) compared to medium and large size cells that did not differ from each other (LSD tests, not significant). Interestingly, among the subpopulation of ganglion cells with high mean somatic staining intensity, medium size cells differed significantly in intensity from small and large size cells (LSD tests, *p* < 0.01), both of which did not show significant difference (LSD test, not significant) from each other.

These different features are also illustrated in the scatter plots of relationship between the mean somatic IR and SD of somatic IR for individual cells (Figures 3B,C). These plots show subpopulations with the distinct neuronal staining patterns identified in Figure 2; lines have been inserted in Figures 3B,C to indicate boundaries that are (1) 1.3 SD units from means of both small and medium sized populations (left line) and (2) 1.5 SD units from the means of both medium and large cell populations (right line). The low IR intensity cell population (Figure 3B) consists largely of cells with low SD, suggesting homogeneous distribution of IR within the soma. The subpopulation of large sized cells in this low IR intensity group correspond to cells illustrated in Figures 2C,D, while the medium and small sized cells are represented in Figures 2G,K. On the other hand, a subpopulation of intensely immunoreactive, punctately stained medium and a few small sized cells (demarcated red square in Figure 3C) has cells with highly variability (inhomogeneous) intra somatic immunoreactivity, thus corresponding to cells represented in Figures 2F,H. Within the population of high IR intensity cells, there were some small sized cells with more homogeneous intense staining. These have a low variability in intrasomatic IR intensity (low SD) and are exemplified in Figure 2I.

### Expression of the 5-HT_1F_ Receptor Subtype in Trigeminal Ganglia

**Figure 4:**
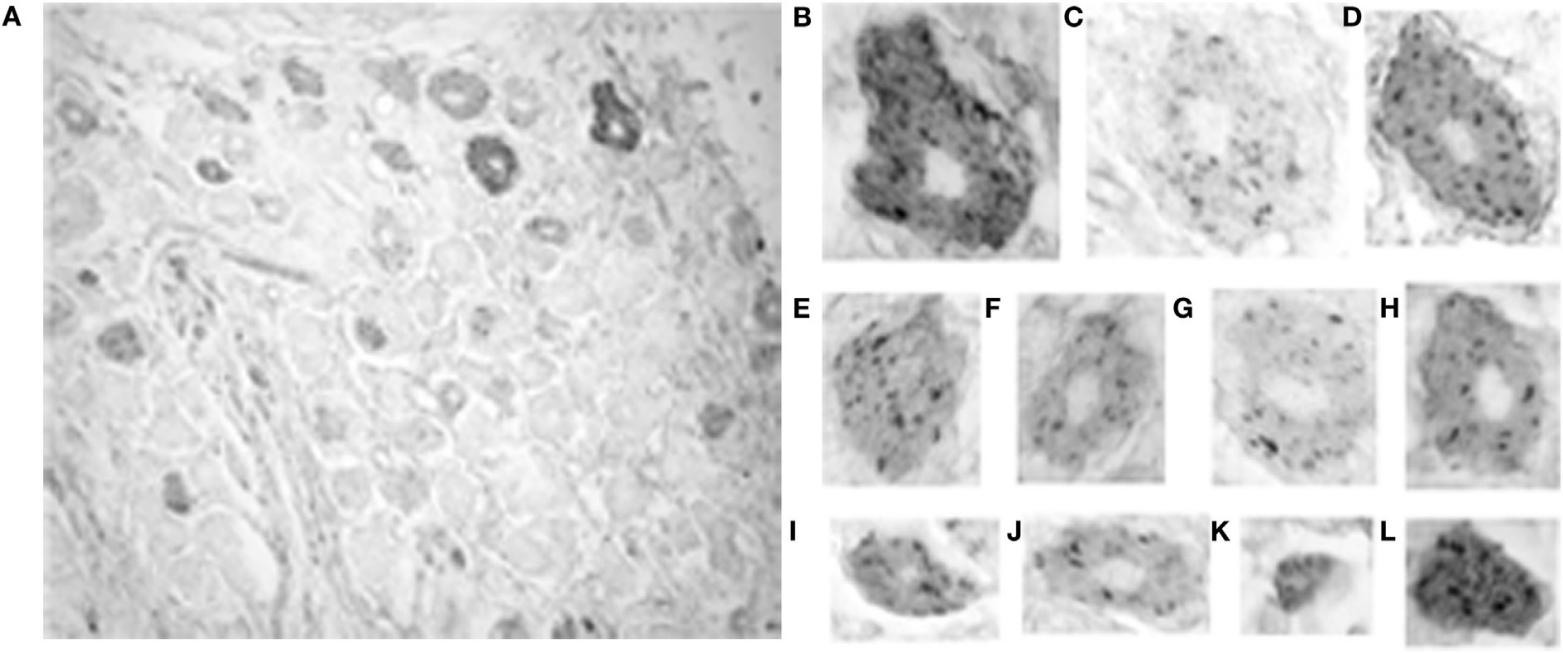
**Photomicrograph of 5-HT_1F_ receptor immunoreactive neurons in the macaque trigeminal ganglia**. **(A)** Notice the presence of considerably fewer immunopositive cells in general and fewer immunopositive large size cells in particular, compared to the vestibular ganglia. The calibration bar represents 40 microns; **(B–L)** Staining patterns and associated cell sizes in representative trigeminal ganglion cells. Most 5-HT_1F_ receptor immunopositive trigeminal ganglia have a similar punctate staining pattern. A few large **(B,D)** and small **(L)** cells showed more intensely stained background cytoplasm.

The intensity of somatic 5-HT_1F_ receptor immunoreactivity was also analyzed qualitatively and quantitatively in monkey trigeminal ganglia. By contrast, the prevalence of immunoreactivity for the 5-HT_1F_ receptor was considerably lower in trigeminal ganglion cells with only 74.2% of neurons showing immunopositivity above background. However, the full range of size categories from small through medium to large cells were represented among positively stained neurons (Figure 4). Unlike 5-HT_1F_ receptor immunopositive vestibular ganglion cells, there was considerably less variability in intensity and cellular distribution among various trigeminal ganglion cells (Figure 4). The majority of 5-HT_1F_ receptor immunopositive trigeminal ganglion cells exhibited a similar punctate staining pattern (Figures 4B–L). In addition, a few large (Figures 4B,D) and small (Figure 4L) trigeminal ganglion cells showed more intense staining in background cytoplasm.

**Figure 5:**
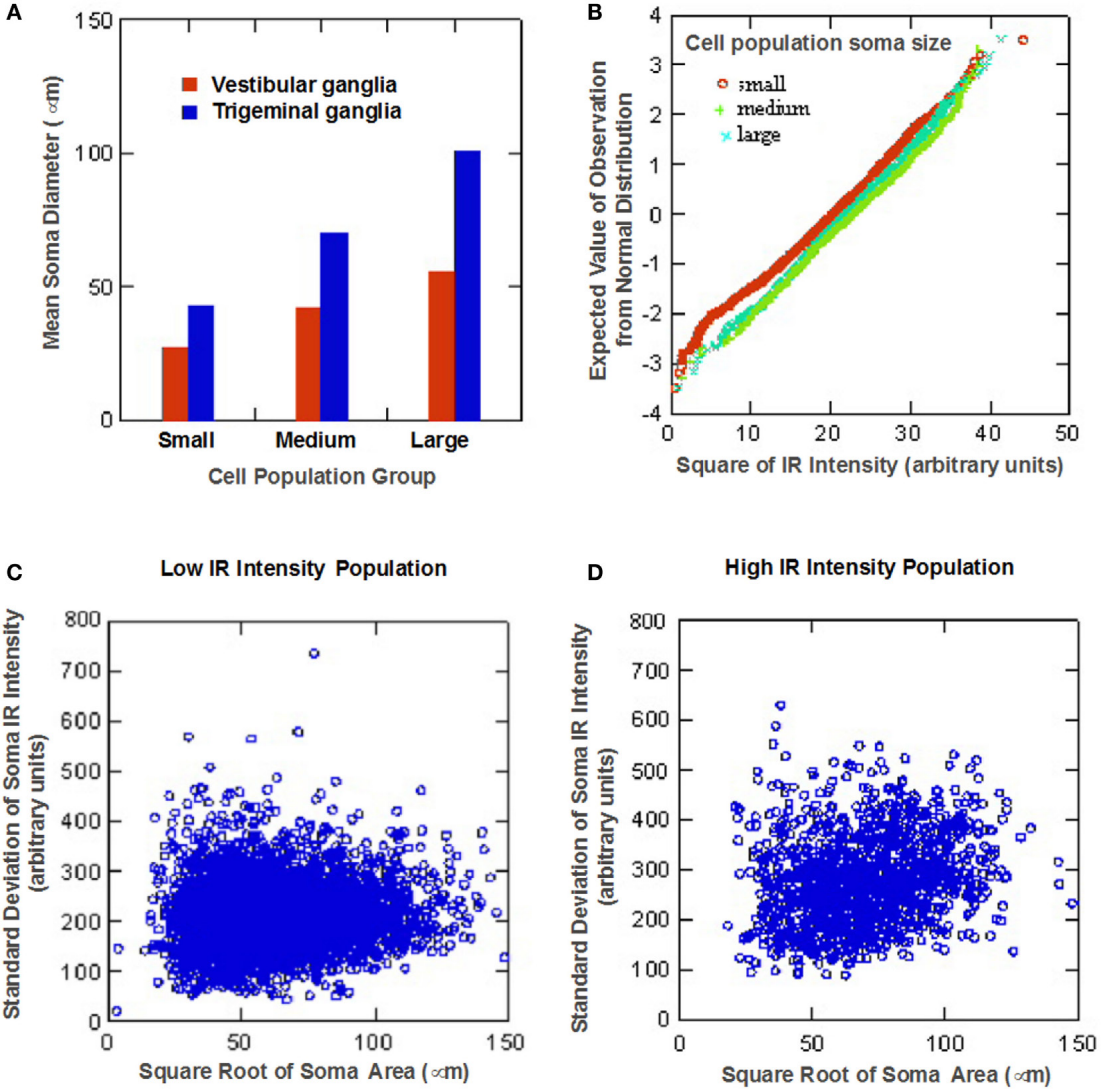
**Statistical analysis of neuronal 5-HT_1F_ receptor immunohistochemistry in trigeminal ganglion cells from monkey temporal bones**. **(A)** Bar graph comparing the identified small, medium, and large sized cell subpopulations shows larger neuronal somatic dimensions for trigeminal ganglion cells compared to vestibular ganglion cell subpopulations; **(B)** Normal probability plots show ganglion cells with higher immunoreactive intensity tend to be larger than lower staining intensity cells, low **(C)** and high **(D)** somatic staining intensity populations identified by cluster analysis of log mean cell staining intensity data show a larger proportion of TG cells fell in the low intensity subpopulation.

Cluster analysis revealed that size distribution of 5-HT_1F_ receptor immunopositive trigeminal ganglion cells similar to immunopositive vestibular ganglion cells is consistent with three normally distributed (Gaussian) populations comprising 38.0% from a 49.5 ± 10.0 (SD) micron average circular diameter population, 42.2% from a population with a mean circular diameter of 79.3 ± 9.3 (SD) micron and 19.8% from a 114.0 ± 13.7 (SD) micron average circular diameter. It is noteworthy that the small, medium, and large sized cell subpopulations identified for trigeminal ganglion cells showed consistently larger neuronal somatic dimensions compared to their vestibular ganglion cell subpopulation counterparts (Figure 5A).

Quantitative analysis of the somatic immunoreactivity intensity of 5-HT_1F_ receptor positive trigeminal ganglia further revealed similarities and additional differences compared to vestibular ganglia. The normal probability plots (Figure 5B) showed that, similar to vestibular ganglion cells, trigeminal ganglion cells with higher immunoreactive intensity tended to be larger than lower staining intensity cells. The scatter plots of the intracellular mean IR versus intracellular SD of low (Figure 5C) and high (Figure 5D) somatic staining intensity populations (identified by cluster analysis of mean cell staining intensity data for trigeminal ganglion cells) showed general similarity in cell size distribution patterns. Although the linear relationship between soma size of the high intensity population and the SD of the somatic IR is statistically significant (*p* < 0.01), it only accounted for 2.3% of the variance. Both subpopulations, however, showed consistently higher values compared to their vestibular ganglion counterparts. Specifically, 49.0% showed a low mean somatic intensity (16.4 ± 4.0, square root arbitrary units), while 51.0% had a higher mean intensity (26.5 ± 3.7, square root arbitrary units).

### Expression of the 5-HT_1F_ Receptor Subtype in the Auditory Periphery

**Figure 6:**
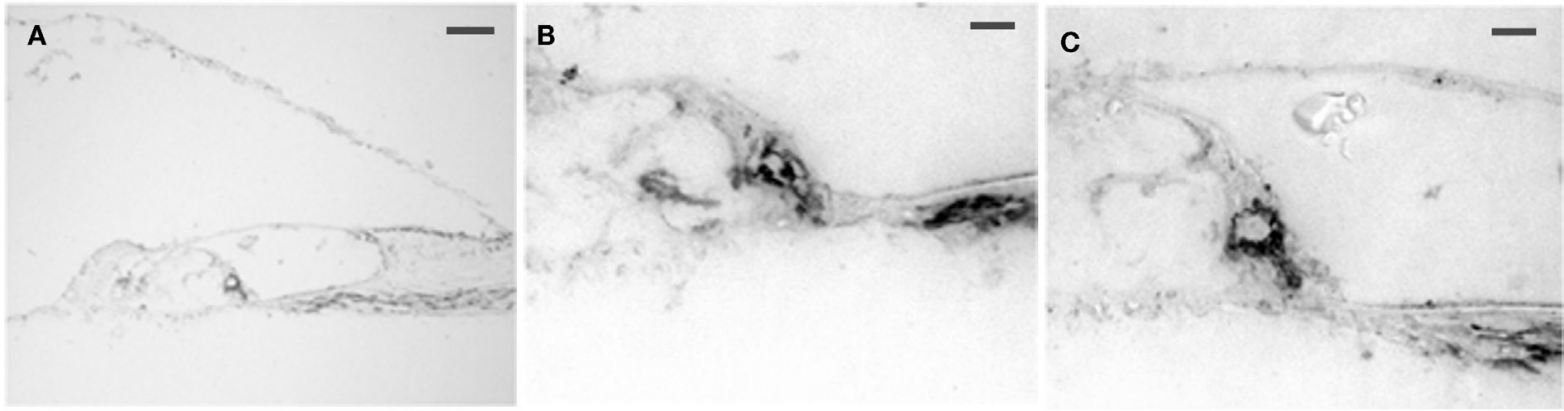
**Photomicrograph of 5-HT_1F_ receptor immunoreactive spiral ganglion cell dendrites in the macaque organ of Corti**. **(A)** The axons could be traced from the spiral ganglion to the base of the inner hair cells. No 5-HT_1F_ receptor immunoreactive axons were associated with the outer hair cells. The calibration bar represents 100 microns. **(B,C)** Examples of staining in association with the inner hair cells are shown below at higher magnification (bar = 20 microns).

**Figure 7:**
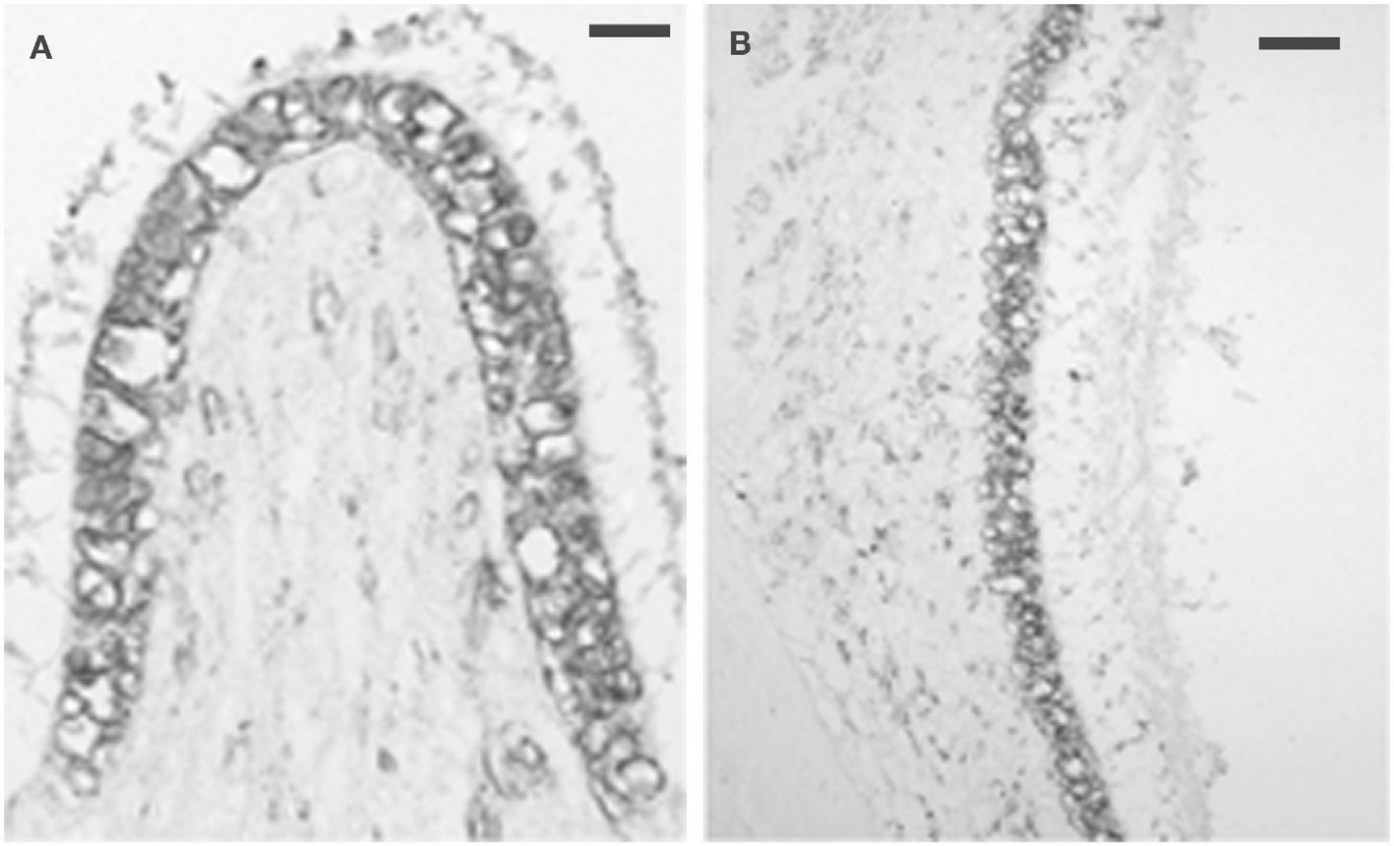
**Photomicrograph of 5-HT_1F_ receptor immunoreactivity associated with both the neurepithelium and blood vessels**. **(A)** Macaque posterior semicircular canal crista (left) and **(B)** saccular macula. The calibration bar represents 60 microns.

Intense 5-HT_1F_ receptor staining is associated with the peripheral afferents of the eighth nerve. In the cochlea, immunopositive axons could be traced distally from spiral ganglion cells to the organ of Corti (Figure 6A). These axons ended in either large, knob-like terminals (Figure 6B) or calyx-like endings (Figures 6A,C) on inner hair cells. No positive axons were seen traversing the tunnel of Corti or at the outer hair cells. In the cristae ampullares (Figure 7A) and maculae (Figure 7B), immunoreactivity was associated with blood vessels and cell bodies of hair cells, where it could appear diffusely or as bouton-like or calyx-like terminations.

## Discussion

In addition to migraine, the complex interplay between the various serotonin receptors in the CNS has been linked to responses to stress, complex cognitive responses, anxiety, and spatial memory. The strong parallels in serotonergic receptor expression between migraine pathways and vestibular pathways (6, 7, 24, 45) further support the notion that 5-HT_1_ receptor subtypes may play a role in modulating signs and symptoms of vestibular migraine. In particular, the higher prevalence of expression of 5-HT_1F_ (and 1B/D receptors) in the vestibular ganglion cells suggests a crucial role for these receptor subtypes.

Previous studies from our laboratory demonstrated that serotonergic 5-HT_1B_ and 5-HT_1D_ receptors are expressed extensively in inner ear ganglion cells of all sizes in monkeys and rats (31). In this study, we describe the distribution in monkey vestibular ganglion cells of the 5-HT_1F_ receptor subunit, another serotonergic receptor subtype that has high affinity for triptans (46, 47). Our results show that 5-HT_1F_ receptor expression in macaque vestibular ganglion cells not only shows obviously different cellular distribution patterns than 5-HT_1B_ and 5-HT_1D_ subtypes, but also differs relative to 5-HT_1F_ receptor subtype in trigeminal ganglion cells. While 5-HT_1B_ and 5-HT_1D_ receptor immunoreactivity have been detected in 97 and 87% of macaque vestibular ganglion cells, respectively (31), 5-HT_1F_ receptor immunoreactivity was present above background in virtually all (99.99%) vestibular ganglion neurons. The appearance of heavy immunoreactivity in association with vestibular hair cells was consistent with contributions from calyceal, bouton, and dimorphic terminals described by others (48–50). Hence, a high level of co-localization between 5-HT_1B_, 5-HT_1D_, and 5-HT_1F_ receptor subunits in vestibular ganglia (on the order of 90%) must be considered as a factor in the efficacy of triptans (and SSRIs) in vestibular migraine (49).

Two main varieties of 5-HT_1B/D/F_ receptor immunohistochemical staining intensity patterns have been identified in trigeminal and dorsal root ganglion cells, which are often associated with cell size. These include staining described as either “dense” granular or less dense “punctate” staining within the cytoplasm and associated mainly with large sized cells (51, 52). Vestibular ganglion cells show a clear association between cell size and somatic immunoreactivity intensity of 5-HT_1F_ receptor immunopositive ganglion cells, which was not seen previously with 5-HT_1B_ and 5-HT_1D_ subunit staining (31). While more intense 5-HT_1F_ receptor somatic immunoreactivity was clearly associated with smaller sized ganglion cells, a population of mainly medium size and a few small size cells with punctate, dense staining previously could be identified quantitatively by a high somatic mean intensity as well as high SD of intensity across the soma. The heterogeneity in staining patterns suggests that different phenotypes of vestibular ganglion cells may be characterized on the basis of both soma size and intracellular serotonin 1 receptor distribution. For example, one expects that both thick calyx afferents, which arise from large, calretinin positive vestibular ganglion cells that show little or no overlap in staining with peripherin, another biochemical marker known to stain bouton-only vestibular afferents (53–59) and afferents arising from “medium” calbindin positive vestibular ganglion cells that show some overlap (48, 57) would have varied distribution in 5-HT_1F_ receptor expression.

The heterogeneity in intracellular 5-HT1 receptor staining patterns may be noteworthy in light of both structural differences in native 5-HT_1_ receptor subtypes and evidence for homo- and hetero-oligomerization. Serotonergic 5-HT_1_ receptors are among the G-protein-coupled receptors (GPCRs) that are known to activate canonical as well as non-canonical second messenger signal pathways. Functional selectivity or biased signaling occurs when “biased” ligands preferentially or differentially activate one signaling pathway over another, with distinct potencies and efficacies; as opposed to unbiased agonists that activate alternative pathways equally (60). Although recent studies have provided evidence of critical structural determinants for 5-HT_1B_ receptor–ligand recognition and subtype selectivity (61), there are no known similar reports on either the 5-HT_1D_ or 5-HT1_1F_ receptor subtypes. While the 5-HT_1B_ and 5-HT_1D_ receptors are closely related on the basis of degree of homology in the sequence alignment (62), they are divergent from the 5-HT_1F_ receptor subtype (63). In addition, there is ample evidence that 5-HT_1B_ and 5-HT_1D_ receptors have the propensity to form homodimers when expressed alone and heterodimers when co-expressed *in vitro* (42, 43). The concept of 5-HT_1B_ and 5-HT_1D_ homodimers and heterodimers opens interesting directions for exploring interactions between co-expressed 5-HT_1B_, 5-HT_1D_ and 5-HT_1F_ receptors. Studies suggest that heterodimerization between 5-HT_1B_ and 5-HT_1D_ receptors may require a co-translational or specific cellular mechanism and appears to be favored over homodimerization when these subtypes are co-expressed (43). Although there is a dearth of information on oligomerization of 5-HT_1F_ receptors, recent evidence indicates that phosphorylation by PKA is involved in regulating the stability of heteromers of dopamine and adenosine G-protein-coupled receptors through interactions at short linear motifs on the receptor subunit proteins (64). Hence, dynamic regulation of heteromerization and oligomerization may be a factor in understanding both fluctuations within and individual and inter individual differences in effects of ligands.

Initial studies of 5-HT_1B_ and 5-HT_1F_ receptor heterodimers showed serotonin binding kinetics intermediate to 5-HT_1B_ and 5-HT_1D_ receptor homodimers, which suggests a similar pharmacology (42, 43). However, data from studies on other related GPRCs, such as GABA_B_ (37–41) and opioid receptors (44), suggest that heterodimerization of GPRCs may result in novel functionality. It is, therefore, a possibility that heterodimerization between 5-HT_1B_ and 5-HT_1D_ receptors may result in greater diversity of 5-HT_1_ receptor function in general and 5-HT_1F_ receptor activity in particular, on account of inter-receptor interactions that maybe potentially modulated by other receptor subtypes. Therefore, experiments targeted at elucidating the possible altering effects of receptor hetero-oligomerization between 5-HT_1B_, 5-HT_1D_, 5-HT_1F_, and indeed other receptor types on functional selectivity in signal transduction would be crucial to understanding receptor–ligand interactions and designing subtype-selective and more effective serotonergic drugs.

The quantitative distribution of 5-HT_1B_, 5-HT_1D_, and 5-HT_1F_ receptor subtypes has been examined previously in rat trigeminal and/or dorsal root ganglion cells (51, 65–68), with co-localized staining primarily in the cytoplasm of immunopositive ganglion cells. More recent studies further demonstrated no apparent differential in both distribution and staining intensity pattern between the 5-HT_1_ receptor subtypes in rat trigeminal and dorsal root ganglia (52), leading the authors to conclude that a simple correlation between the level of expression of these receptors in nociceptive ganglia (trigeminal and dorsal root ganglia) is not sufficient to explain the efficacy of triptans in migraine and cluster headaches. In the case of macaque trigeminal ganglion cells in our study, expression of 5-HT_1F_ receptor immunoreactivity was primarily in small and medium sized neurons with a more limited number of large sized ganglion cells showing immunopositivity.

These findings corroborate previous observations made for cell size distribution for 5-HT_1F_ immunopositive cells in rat trigeminal ganglia (16, 69, 70). Interestingly, Ma (2001) reported that 5-HT_1F_ receptor expression in rat trigeminal ganglia was largely in small diameter cells, while our results in monkeys showed small portion of 5-HT_1F_ receptor immunopositive large diameter cells in addition to small and medium sized cells. Inter-species variations in receptor expression patterns are far from unprecedented, as was evident in our previous studies showing differential association between intensity of expression of 5-HT_1B_ and _1D_ receptors in rat and monkey ganglion cells (Ahn and Balaban, 2011). It is, therefore, not surprising that quantitative expression of immunopositive cell size populations seem to vary slightly between rat and monkeys. However, our other related discoveries highlight several distinctions in quantitative and qualitative expression of 5-HT_1F_ receptor immunoreactivity between macaque vestibular and trigeminal ganglion cells. First, a lower proportion (74.2%) of trigeminal ganglion cells was 5-HT_1F_ receptor immunoreactive. Second, there was considerably less variability in somatic intensity staining and cellular distribution among 5-HT_1F_ receptor immunopositive trigeminal ganglion cells with the majority exhibiting similar punctate staining patterns. Finally, trigeminal ganglion cells showed higher mean somatic immunoreactive intensity and neuronal size proportions for the three size distribution subpopulation identified (small, medium, and large sized cells) were larger compared to vestibular ganglion cell size distribution subpopulations. For instance, the small and medium sized trigeminal ganglion cell subpopulations showed similar somatic size proportions with medium and large vestibular ganglion cells subpopulations, respectively.

Agonists at 5-HT_1F_ receptors have been purported to relieve migraine possibly *via* a number of mechanisms unrelated to vasoconstriction. These mechanisms include blockade of neurogenic plasma protein extravasation (46, 71, 72), inhibition of c-fos expression (73, 74) and inhibition of neuronal firing (75) in the trigeminal nucleus caudalis. Furthermore, inhibition of plasma protein extravasation by serotonin agonists has been correlated specifically with activity at 5-HT_1F_ (46) and not 5-HT_1B_ or 5-HT_1D_ receptor subtypes (76, 77) further corroborating possible alternate but complimentary mechanisms of action for these triptan sensitive receptor subtypes. Indeed, these alternative complimentary mechanisms are likely in operation in the vestibular periphery. Koo and Balaban (78) demonstrated that intravenous serotonin infusions can elicit plasma extravasation in specific inner ear tissues. The widespread distribution of serotonin-induced plasma extravasation in the apical spiral ganglion, modiolus as well as intralabyrinthine superior and inferior vestibular nerve provided a “proof-of-concept” that concurrent inner ear and meningeal extravasation could to vestibular symptoms like vertigo and sound sensitivity associated with migraine.

Interestingly, differences in distribution of 5-HT_1F_ versus 5-HT_1D_ in the vestibular periphery are also corroborated by our results. While there is evidence of differential expression of 5-HT_1D_ and _1F_ receptors in planum semilunatum cells as well as vasculature in the crista ampullaris (24), our studies showed that immunoreactivity for 5-HT_1F_ receptors was closely associated with a vestibular and spiral ganglion cells. Our findings further corroborate the notion that variations in 5-HT_1F_ and _1D_ receptor subtype expression in vestibular organs may contribute to mediating multiple influences of triptans on these organs. In addition, similar to what has been speculated for the vestibular ganglion, the phenomena of possible receptor oligomerization between 5-HT1 receptors subtypes in the vestibular periphery may also further contribute to determining the downstream signaling pathway cascades ultimately triggered.

## Author Contributions

Design, conduct of study, analysis, and manuscript preparation.

## Conflict of Interest Statement

The authors declare that the research was conducted in the absence of any commercial or financial relationships that could be construed as a potential conflict of interest.
